# Editorial: T and B cell immune dynamics in allergic response

**DOI:** 10.3389/falgy.2025.1558724

**Published:** 2025-02-12

**Authors:** Mrinmoy Das, Tarun Keswani

**Affiliations:** ^1^ Division of Immunology, Boston Children’s Hospital and Department of Pediatrics Harvard Medical School, Boston, MA, United States; ^2^ Center for Immunology and Inflammatory Diseases, Division of Rheumatology, Allergy, and Immunology, Massachusetts General Hospital and Harvard Medical School, Boston, MA, United States

**Keywords:** allergy, T cell, B cells, inflammation, IgA, IgG, IgE, immune response

**Editorial on the Research Topic**
T and B cell immune dynamics in allergic response

Globally, Allergic diseases poses a significant public health threat, affecting millions worldwide (1). Although considerable progress has been made in allergy research, there is still a pressing need for: Effective, definitive treatments to manage allergic diseases and a deeper understanding of the complex mechanisms governing T and B cell responses in allergic reactions. Elucidating the underlying immunological processes will be crucial for developing innovative, targeted therapies to improve patient outcomes and quality of life.

Allergic diseases occur when the immune system mounts an exaggerated response to typically harmless environmental substance, such as aeroallergens and food proteins. This overreaction is driven by the components of the innate and adaptive immune responses, such as innate lymphoid cells type 2 (ILC2) mast cells, basophils, eosinophils, as well as activated T helper type 2 (Th2) cells and B cells that have switched to the production of immunoglobulin type E (IgE). Immune regulatory mechanisms play a crucial role in maintaining tolerance to allergens and preventing allergic reactions. Genetic and immunological evidence also reinforce the idea of a pivotal role for FOXP3 expressing regulatory T (Treg) cells in promoting tolerance to allergens and preventing allergic disorder (2). T regulatory type (Tr1) cells represent a distinct population of T cells that do not constitutively express FOXP3 and are induced in the periphery upon antigen exposure under tolerogenic conditions. Tr1 cells modulate allergic reactions by releasing IL-10 and TGF-β, but their role is not fully understood (3). Further research on Tr1 cells could lead to new allergy treatments. A large body of evidence shows that B cells are key players in allergic responses, primarily through the production of IgE antibodies that trigger allergic reactions. In addition to their pivotal role in the pathogenesis of allergies, B cells are also involved in regulation of immune response through antigen presentation, producing IgG and IgA antibodies, immunosuppressive cytokines such as IL-10, IL-35 and TGF-β. By understanding the biology of B cells, including their development, types, and functions, researchers can uncover new ways to promote immune tolerance and treat allergic diseases (4). This editorial will summarize recent efforts that have been published in the research topic T and B Cell Immune Dynamics in Allergic Response in the journal Frontiers in Allergy.

Researchers led by Pablo-Torres et al., uncovered a link between severe allergies, metabolic problems, and impaired immune function. Their study revealed that severe allergic asthma patients have distinct T-cell gene expression profiles, marked by metabolic and immune pathways disruptions, reduced energy production, and increased inflammation. Specifically, genes involved in energy production (oxidative phosphorylation, fatty acid oxidation, and glycolysis) were downregulated, while genes coding for inflammatory cytokines (IL-19, IL-23A, and IL-31) were upregulated. Additionally, genes involved in TGF-β pathway, as well as Tregs percentage were downregulated indicating impaired regulatory function in these cohorts of severe allergic asthmatic patients. Notably, in cases of food allergy, the deletion of TGF-β1 in Tregs impaired the differentiation of RORγt + Treg cells, leading to mast cell expansion in the gut and increased susceptibility to food allergy development (5). These findings shed new light on the complex relationships between immune regulation, metabolism, and severe allergic inflammation, paving the way for novel therapeutic approaches.

Stichova et al., investigated the function of CD46's, particularly in allergic eosinophilic asthma, and the influence of calcitriol (vitamin D). The study revealed: CD46 plays a critical role in regulating immune cell responses. It regulates the switch from Th1 cells, which produce IFN-γ, to Tr1 cells, known for IL-10 production, which is crucial for balancing immune responses. Vitamin D (calcitrol) enhances immune regulation by promoting specific immune cells (6). Research has shed light on the crucial role of TR1 cells in regulating immune responses in asthma. By promoting TR1 cell development and increasing IL-10 levels, scientists may have uncovered a promising therapeutic strategy for managing asthma. Eosinophils contribute to asthma inflammation through CD46. Elevated eosinophil cationic protein (ECP) levels during pollen exposure impact CD46 expression and influence T cell responses in AEA (7). In addition, these interactions during clinical studies may contribute to understanding asthma pathophysiology, including exploring vitamin D supplementation to and regulate inflammation.

Researchers Furiness et al. reviewed the therapeutic potential of IgA and IgG antibodies specific to allergens in food allergy management. These antibodies may counterbalance the severe reactions triggered by IgE antibodies by inhibiting mast cell activation, offering a promising approach to preventing and alleviating allergy symptoms through oral immunotherapy. Although advancements in diagnosing and managing food allergies have been made, no curative treatments are currently available. Recent findings from oral Immunotherapy studies suggest that IgA and IgG antibodies specific to allergens might inhibit the effects of IgE on mast cells. The review highlights the dual role of mast cells in food allergies: they are responsible for immediate allergic reactions and help develop adaptive immune responses, particularly those driven by Th2 cells. These antibodies may regulate the immune system, decrease allergic responses, and block pathways that lead to allergy symptoms. A more comprehensive understanding of the regulatory role of IgA and IgG in food allergy may provide insights into physiologic regulation of immune responses to ingested antigens and could seed novel strategies to mitigate allergic disease (8).

A rare medical condition, insulin-induced Type III hypersensitivity reactions (HSRs), is characterized by immune complexes formed by insulin and IgG antibodies, leading to symptoms like subcutaneous nodules and severe insulin resistance. An extensive review by Meredith et al. of 16 similar cases reveals that Type 1 diabetes patients tend to have a more aggressive disease course compared to Type 2 patients, and while immunosuppressive therapy provides temporary relief, its long-term benefits are uncertain, warranting further research. Adding to these discoveries, a case study of a 43-year-old woman with Type 1 diabetes mellitus highlights the challenges of diagnosing and managing this condition. Meredith et al., demonstrated the treatment approaches, including therapeutic plasma exchange and immunosuppression, but long-term effectiveness is uncertain. Further research is necessary to understand insulin-induced Type III HSRs mechanisms and develop consistent treatment guidelines.

Recent studies Integrating the key players in immune regulation, including Tr1 cells, B cells, IgA, IgG, and CD46, holds promise for addressing the unmet clinical needs in allergy research. Furthermore, these contributors work in concert to modulate immune responses, maintain tolerance, and prevent excessive inflammation. Elucidating the interplay between these key contributors may lead to the development of novel therapeutic strategies, improved diagnostics, and enhanced understanding of allergic diseases. The studies offer groundbreaking insights and potential pathways for developing novel, innovative therapies to combat allergies, asthma, and autoimmune diseases. These studies have far-reaching implications, offering hope for millions of people worldwide affected by allergies, asthma, and autoimmune diseases.
